# Cerebrospinal Fluid Levels of Amyloid Beta 1–43 in Patients with Amnestic Mild Cognitive Impairment or Early Alzheimer’s Disease: A 2-Year Follow-Up Study

**DOI:** 10.3389/fnagi.2016.00030

**Published:** 2016-03-01

**Authors:** Camilla Lauridsen, Sigrid B. Sando, Adiba Shabnam, Ina Møller, Guro Berge, Gøril R. Grøntvedt, Inger J. Bakken, Øyvind Salvesen, Geir Bråthen, Linda R. White

**Affiliations:** ^1^Department of Neuroscience, Faculty of Medicine, Norwegian University of Science and TechnologyTrondheim, Norway; ^2^Department of Neurology, University Hospital of TrondheimTrondheim, Norway; ^3^Norwegian Institute of Public HealthOslo, Norway; ^4^Unit for Applied Clinical Research, Faculty of Medicine, Norwegian University of Science and TechnologyTrondheim, Norway

**Keywords:** Alzheimer’s disease, amnestic mild cognitive impairment, biomarkers, amyloid beta 1–43, amyloid beta 1–42, cerebrospinal fluid, diagnostic accuracy

## Abstract

**Introduction:** Biomarkers that will reliably predict the onset of Alzheimer’s disease (AD) are urgently needed. Although cerebrospinal fluid (CSF) amyloid beta 1–42 (Aβ42), total tau, and phosphorylated tau can be used to complement the clinical diagnosis of AD, amnestic mild cognitive impairment (aMCI), the prodromal phase of AD, is heterogeneous. Biomarkers should be able to determine which patients with aMCI are at greatest risk of AD. Histological studies and animal models indicate that amyloid beta 1–43 (Aβ43) aggregates early, and may play a role in the pathological process of AD. We have examined levels of CSF Aβ43 in a 2-year longitudinal study of aMCI and early AD.

**Materials and Methods:** Cerebrospinal fluid was collected at baseline, and after one and 2 years from patients with AD (*n* = 19), and patients with aMCI (*n* = 42). Of these, 21 progressed to AD during the 2 years of study, whereas 21 did not. Controls (*n* = 32) were lumbar punctured at baseline only. CSF analyses of Aβ43, Aβ42, and total tau were carried out with ELISA.

**Results:** At baseline, CSF Aβ43, CSF Aβ42 and ratios with total tau could be used to separate controls from all three patient groups. CSF Aβ43, but not Aβ42, could separate patients with aMCI who progressed to AD during the 2 years of follow-up, from those that did not. The CSF total tau/Aβ43 ratio had a slightly but significantly larger area under the receiver operating characteristic curve when compared to the CSF total tau/Aβ42 ratio. CSF Aβ43 levels, but not Aβ42 levels, decreased from baseline to 2 years in the AD group.

**Discussion and Conclusion:** CSF Aβ43 was demonstrated to be significantly reduced in patients already by the time that aMCI or AD was diagnosed, compared to controls, and this change must have occurred during the preclinical period. Since our results suggested that CSF Aβ43 distinguishes between subgroups of patients with aMCI better than CSF Aβ42, it may prove to be a useful additional biomarker for identifying aMCI patients at greatest risk of AD.

## Introduction

According to the proposed new diagnostic criteria for Alzheimer’s disease (AD), early AD can be diagnosed if the prodromal phase (amnestic mild cognitive impairment, aMCI) is present, and at least one biomarker is positive, though these biomarkers have yet to be validated (Albert et al., 2011; McKhann et al., 2011; Dubois et al., 2014). Biomarkers with high sensitivity and specificity that reflect the underlying pathology of AD will therefore be important in identifying patients with aMCI who are most likely to progress to AD. Such identification will be crucial when efficient treatment becomes available.

Levels of amyloid beta 1–42 (Aβ42) in cerebrospinal fluid (CSF) correlate inversely with brain parenchymal Aβ42 load, as visualized by positron emission tomography (PET) (Fagan et al., 2006; Tolboom et al., 2009). Additionally, CSF Aβ42 levels have repeatedly been found to be reduced in patients with MCI and AD, compared to healthy controls (Sunderland et al., 2003; Hansson et al., 2006). CSF Aβ42 levels are usually reduced years before clinical symptoms of dementia appear (Buchhave et al., 2012), and CSF Aβ42 is therefore considered a candidate biomarker for early AD, alone or in combination with other CSF biomarkers such as total tau (t-tau) representing axonal degeneration, or phosphorylated tau representing tau hyperphosphorylation (Hansson et al., 2006; Tapiola et al., 2009). The combination of CSF Aβ42 and t-tau has been found to predict incipient AD with a sensitivity ≥88% and specificity ≥82% (Hansson et al., 2006; Hertze et al., 2010), while CSF Aβ42 alone predicted AD in patients with MCI with a sensitivity of 79% and specificity of 65% (Mattsson et al., 2009).

Compared to Aβ42, amyloid beta 1–43 (Aβ43) has an additional threonine at the C-terminal, and is thought to be more aggregation-prone than Aβ42 (Jarrett et al., 1993; Saito et al., 2011; Conicella and Fawzi, 2014). Aβ43 was found to be the first amyloid beta peptide to deposit in mutant amyloid precursor protein transgenic mice (Zou et al., 2013), and Aβ43 was theorized to provide a ‘seed’ for subsequent Aβ42 deposition (Conicella and Fawzi, 2014), suggesting that both peptides are likely to be involved in early plaque formation (Jarrett et al., 1993; Parvathy et al., 2001). Aβ43 may therefore play a role in AD pathogenesis despite its low level in brain tissue (Sandebring et al., 2013), as it has been shown to have equal or even greater neurotoxicity than Aβ42 in PS1-R278I knock-in mice (Saito et al., 2011). In a study from human brain tissue, Aβ43 was frequently found in plaques, and no amyloid peptides longer than Aβ43 were found (Welander et al., 2009).

To date there are few studies assessing CSF Aβ43 for its potential as a biomarker in early AD. It has been demonstrated that CSF Aβ43 was decreased in MCI and AD patients, as well as being positively correlated to CSF Aβ42 (Kakuda et al., 2012), despite Aβ43 and Aβ42 being produced by different enzymic routes (Qi-Takahara et al., 2005). CSF Aβ43 was found to be slightly inferior to Aβ42 for separating controls from AD patients (Bruggink et al., 2013).

In the present study, we examined CSF Aβ43 levels at baseline, and longitudinal levels over a 2-year period, in samples from patients with aMCI that progressed to AD during this period, as opposed to those that did not. Levels in patients with AD from baseline were also studied, and results for healthy control individuals were available at baseline. Comparisons were made with CSF levels of Aβ42, including correlations, and ratios with CSF t-tau.

## Materials and Methods

### Subjects

Patients were recruited through the Department of Neurology, St. Olav’s Hospital (University Hospital of Trondheim) and assigned to this study from 2009 onward. Patients were referred to the clinic by general practitioners, and were diagnosed by a neurologist (SBS). Patients with early AD (*n* = 19) were diagnosed according to the NINCDS–ADRDA criteria (McKhann et al., 1984), and patients with aMCI (*n* = 42) according to the International Working Group on Mild Cognitive Impairment Criteria (Winblad et al., 2004). Patients were followed over a 2-year period. During this time, 21 patients diagnosed with aMCI progressed to AD. This subgroup is subsequently designated “pMCI” in the results, for convenience. The remaining 21 patients with aMCI did not progress to AD, and are similarly designated “sMCI”. All patients were ethnic Norwegians, with sufficient sight and hearing to complete the cognitive tests. Exclusion criteria were a present psychiatric or malignant disease, use of anti-coagulating medication, or high alcohol consumption.

Thirty-two elderly volunteers were recruited as controls from societies for retired people in central Norway, or were caregivers not genetically related to the patient. These control individuals were also examined by SBS and were healthy for their age without signs of a neurological disorder. Four had first-degree relatives with dementia. Controls were examined at baseline and after 2 years, but CSF samples were obtained only at baseline.

The neurological examination performed on both patients and controls included the Mini Mental State Examination (MMSE) (Folstein et al., 1975), as well as cerebral MRI at 3T, at baseline, and after 2 years. Apolipoprotein E (*APOE*) genotyping was performed on blood samples from all study participants as described elsewhere (Berge et al., 2014). Diagnostic assessment and biomarker analysis were independent of each other. The demographic data are shown in **Table 1**.

**Table 1:** Demographic data.

		aMCI at baseline		
	Controls	sMCI	pMCI	AD at baseline
Individuals included (n)	32	21	21	19
Gender (% females)	75.0	52.4	57.1	47.4
Age at inclusion (years)	67.3 ± 3.7	65.5 ± 6.4	63.8 ± 4.3	65.6 ± 6.1
Age at onset (years)	N/A	63.0 ± 7.1	61.1 ± 4.2	62.4 ± 6.2
Duration of symptoms (years)	N/A	2.5 ± 1.5	2.8 ± 1.0	3.2 ± 1.9
% *APOE* 𝜀4 carriers	31.0	47.6	76.2**^#^**	84.2**^##^**
Education (years)	13.7 ± 3.2	12.9 ± 3.6	13.2 ± 3.7	12.1 ± 3.8
MMSE score at baseline	29.5 ± 0.7^∗^	28.0 ± 1.4^∗^	26.6 ± 1.9^∗^	22.9 ± 2.9^∗^
after 1 year	N/A	28.1 ± 1.3	24.5 ± 2.5∗∗	19.4 ± 4.2∗∗, *n* = 18
after 2 years	N/A	28.1 ± 1.3	22.5 ± 2.8∗∗, *n* = 20	16.9 ± 5.3∗∗, *n* = 14

*Results for continuous variables are given as the mean ± SD. **^#^**Significantly different to the control group (*p* = 0.002). **^##^**Significantly different to the control group (*p* < 0.001) and the sMCI group (*p* = 0.015).^∗^ Significant difference between all groups at baseline (all pairwise comparisons: *p* ≤ 0.010).∗∗ Significantly reduced levels compared to baseline (*p* ≤ 0.001). AD: Alzheimer’s disease, aMCI: amnestic mild cognitive impairment, sMCI: patients with mild cognitive impairment that did not progress to AD over 2 years, pMCI: patients with mild cognitive impairment that progressed to AD over 2 years, *APOE* 𝜀4, apolipoprotein E gene 𝜀4 allele; MMSE, Mini Mental State Examination; N/A, not applicable.*

### Sampling of CSF

Cerebrospinal fluid was usually collected early in the morning with patients lying on their side, and the puncture was at the L4/L5 or L5/S1 level. The first 2.5 mL CSF was used for routine clinical investigation. Aliquots of CSF (1 mL) were then collected directly into 2 mL polypropylene cryovials (Corning) immersed in ice-water. Samples were not centrifuged unless contaminated by blood (four samples), and were frozen within 30 min of lumbar puncture, and stored at -80°C until analysis.

### ELISA Assays

Cerebrospinal fluid was analyzed using ELISA monoplex kits [Aβ43 (IBL, cat. no. 27710), Aβ42 (Innogenetics, cat. no. 80324), and total tau protein (Innogenetics, cat. no. 80323)]. Cross-reactivity for Aβ42 in the Aβ43 ELISA was given as <1%. Although this would contribute slightly to measurements for Aβ43, it would be a constant for both control and patient groups. Aβ43 was reported to have 50x less affinity than Aβ42 for the antibodies in the Aβ42 kit.

Samples were thawed in ice-water prior to analysis, and kits were run according to the manufacturers’ instructions. No samples underwent more than one freeze-thaw cycle, and all were analyzed undiluted, in duplicate. Both control and patient samples were included on each plate, and samples from patients in the three groups were evenly distributed across the plates. For the Aβ43 assay, an internal control was run on 5 of 6 plates, yielding an inter-assay CV of 14.4%, and an intra-assay CV of 5.1%.

### Statistical Analysis

Statistical analyses were carried out using SPSS version 22 (IBM), Stata version 13.1., and R version 2.13.1. For all analyses, *p*-values <0.05 were considered statistically significant.

Demographic data and MMSE scores at baseline were assessed using Pearson’s chi-square test for dichotomous variables, or one-way ANOVA for continuous variables, followed by the least-significant difference (LSD) *post hoc* test for pairwise comparisons if significant *p*-values were obtained. Longitudinal MMSE-scores were analyzed in a mixed linear model. Biomarker concentrations at baseline, after 1 year and after 2 years, were log-transformed to the natural logarithm (ln) to approximate to a normal distribution before comparison of groups by one-way ANOVA, followed by the LSD *post hoc* test for pairwise group comparisons. Pearson’s correlation coefficient (*r*) for correlations between biomarkers was also calculated from log-transformed values.

Receiver operating characteristic (ROC) curves were made to assess the diagnostic accuracy for individual biomarkers, or combinations of them, and the area under each ROC curve (AUC) was calculated.

Youden’s index [(sensitivity + specificity)-1] was determined to find exploratory cut-offs (not shown) where the sum of sensitivity and specificity was maximized, for biomarkers or ratios of biomarkers. Pairwise comparisons of AUC between Aβ43 and Aβ42, or ratios including these two biomarkers and total tau protein, were made for pairs of diagnostic groups (DeLong method). Longitudinal data have been analyzed using a mixed linear model. To account for repeated measurements, de-identified patient ID was included as a random effect. The combination of group and time was included as a fixed effect. All biomarker concentrations were log-transformed to the natural logarithm (ln) to approximate to a normal distribution before analysis in the mixed linear model.

Possible confounding factors included age, gender, and *APOE* genotype. Age and gender were found not to be confounding factors in the present study, and correction for *APOE* genotype would have resulted in many small groups, so was not done. There are results indicating that CSF Aβ42 levels reflect amyloid deposition in the brain independent of *APOE* 𝜀4 status (Lautner et al., 2014), suggesting no need for different cut-offs for CSF Aβ42 based on *APOE* 𝜀4 status (Molinuevo et al., 2014).

### Ethics

The study was conducted according to the Helsinki Declaration. Written, informed consent was obtained from all patients or suitable proxies, and from all control individuals. The biobank is licensed by the Norwegian Directorate for Health Affairs, and the research project was approved by the Regional Committee for Medical Research Ethics, as well as by the Norwegian National Committees for Medical Research Ethics (approval 2013/150).

## Results

### Demographic Data

Demographic data are summarized in **Table 1**. There were no significant differences in gender distribution between the four participant groups (overall *p* = 0.183), despite a preponderance of females in the control group. Neither the age at inclusion nor the duration of education was significantly different between the four groups. Moreover, no significant difference in the age at onset of symptoms, or duration of symptoms was found between the patient groups.

There was a higher prevalence of the *APOE* 𝜀4 allele in the pMCI subgroup, and amongst patients with AD, compared to the control group (both *p* ≤ 0.002). There was also a higher prevalence of the *APOE* 𝜀4 allele in the AD group compared to the sMCI subgroup (*p* = 0.015). The distribution of *APOE* 𝜀4 alleles was not significantly different between controls and the sMCI subgroup, between patients with AD and the pMCI subgroup, or between sMCI and pMCI subgroups.

At baseline, MMSE-scores varied between all four participant groups (all pairwise comparisons: *p* ≤ 0.010). There were decreases in MMSE-scores from baseline to 2 years in the pMCI and AD groups (both *p* < 0.001), but not in the sMCI group.

### Biomarker Data at Baseline and Diagnostic Accuracy

Means and standard deviations of CSF Aβ43, Aβ42, t-tau/Aβ43, and t-tau/Aβ42 in all groups are shown in **Table 2**. Means and error bars in all groups at baseline are shown graphically in Supplementary Figures S1A–S1D.

**Table 2:** Cerebrospinal fluid (CSF) biomarker data.

			aMCI atbaseline					
	Controls	*n*	sMCI	*n*	pMCI	*n*	AD	*n*
Aβ43 (pg/ml) at baseline after 1 year after 2 years	44.6 ± 13.1# N/A N/A	32	32.0 ± 23.6 31.9 ± 22.1 31.4 ± 22.0	20 20 20	20.0 ± 12.4∗∗ 17.8 ± 8.1∗∗ 18.7 ± 9.4^∗^	21 20 20	18.8 ± 7.5∗∗ 17.0 ± 6.6∗∗ 16.7 ± 7.1∗∗**E**	18 19 15
Aβ42 (pg/ml) at baseline after 1 year after 2 years	1065.5 ± 273.1# N/A N/A	25	663.3 ± 348.6 622.4 ± 355.3 631.4 ± 373.5	20 18 18	528.5 ± 178.3 512.3 ± 175.3 529.7 ± 199.3	20 19 19	475.7 ± 169.8 443.2 ± 155.2 476.7 ± 167.1	15 14 14
t-tau/Aβ43 at baseline after 1 year after 2 years	7.5 ± 7.6# N/A N/A	24	17.3 ± 16.0 17.9 ± 16.9 21.4 ± 22.4 **A**	19 17 18	47.6 ± 40.0^∗∗∗^ 60.1 ± 53.3^∗∗∗^ 70.9 ± 89.3^∗∗∗^**B**	20 18 19	45.1 ± 25.3^∗∗∗^ 49.5 ± 23.9^∗∗∗^ 47.8 ± 37.9^∗^	14 14 11
t-tau/Aβ42 at baseline after 1 year after 2 years	0.35 ± 0.47# N/A N/A	23	0.85 ± 1.00 0.91 ± 1.00 1.12 ± 1.47 **C**	20 18 18	1.70 ± 1.76∗∗ 2.04 ± 2.22∗∗ 2.61 ± 4.36^∗^ **D**	20 19 19	1.88 ± 1.65^∗∗∗^ 1.96 ± 1.62∗∗ 1.55 ± 0.72^∗^	15 14 14

*Results are given as the mean ± SD. Significant group differences (analyzed with ANOVA, followed by LSD *post hoc* test, of log-transformed biomarker data): **#** Control group levels at baseline were significantly different from all three patient groups (all *p* ≤ 0.007). ^∗^Significantly different from the sMCI group (*p* < 0.05). ∗∗Significantly different from the sMCI group (*p* < 0.01). ^∗∗∗^Significantly different from the sMCI group (*p* ≤ 0.001). A graphic representation of the data is given in Supplementary Figures S1A–S1D. Longitudinal analyses (mixed linear model) of log-transformed biomarker data: increased from baseline to 2 years: **A** (*p* = 0.002), **B** (*p* = 0.002), **C** (*p* < 0.001), **D** (*p* = 0.003). Decreased from baseline to 2 years: **E** (*p* = 0.003). AD, Alzheimer’s disease; aMCI, amnestic mild cognitive impairment; sMCI, patients with mild cognitive impairment that did not progress to AD over 2 years; pMCI, patients with mild cognitive impairment that progressed to AD over 2 years; Aβ43, amyloid beta 1–43; Aβ42, amyloid beta 1–42; t-tau, total tau; N/A, not applicable.*

Overall, all four analytes or ratios varied between groups at baseline (all *p* < 0.001). The CSF Aβ43 level in the control group was higher than in any patient group (all *p* < 0.001). The control group was also significantly different from all patient groups for the other three analytes or ratios (all *p* ≤ 0.007). There were differences between AD and the sMCI subgroup for Aβ43 and the t-tau/Aβ43 and t-tau/Aβ42 ratios (all *p* ≤ 0.009), but for Aβ42 there was only a weak trend (*p* = 0.086). Aβ43 and the t-tau/Aβ43 and t-tau/Aβ42 ratios varied between the sMCI and pMCI subgroups (all *p* ≤ 0.008), but no difference for Aβ42 levels was found. No significant differences were found between patients with AD and the pMCI subgroup for any analytes or ratios.

Cerebrospinal fluid Aβ43 and Aβ42 were correlated in all four participant groups at baseline (all *p* ≤ 0.004, *r* = 0.621–0.853), as were t-tau/Aβ43 and t-tau/Aβ42 (all *p* < 0.001, *r* = 0.903–0.959). After 1 year, Aβ43 and Aβ42 were still correlated in the sMCI and pMCI groups (both *p* ≤ 0.001, *r* = 0.736–0.912), but not the AD group. After 2 years, Aβ43 and Aβ42 were correlated in all three patient groups (all *p* ≤ 0.019, *r* = 0.690–0.868). T-tau/Aβ43 and t-tau/Aβ42 were correlated in all three patient groups both after 1 year and after 2 years (all *p* < 0.001, *r* = 0.847–0.969).

Receiver operating characteristic plots (not shown) were created for baseline levels of biomarkers or ratios thereof, and statistics were derived for pairwise comparisons of groups (**Table 3**). Both CSF Aβ43 and Aβ42 showed similar specificity in distinguishing control individuals from patients with AD, but the sensitivity was slightly higher for Aβ43. CSF Aβ43 and Aβ42 were also excellent at separating controls from patients in the pMCI subgroup, with AUCs over 0.90. Sensitivity and specificity were all over 90%, except for slightly lower sensitivity of Aβ42. For separating controls from patients in the sMCI subgroup, all AUC values were over 0.7, and specificity was high (87–100%), whereas sensitivity was much lower (53–74%). On the other hand, the sMCI subgroup was well separated from AD patients by the t-tau/Aβ43-ratio, with a slightly higher sensitivity than the corresponding t-tau/Aβ42-ratio. Aβ43 alone also separated these two groups (AUC 0.73, *p* = 0.024), while Aβ42 alone was not significant. The pMCI and AD patient groups were not separated significantly by any of the biomarkers or ratios thereof. However, the sMCI and pMCI subgroups were separated by ratios t-tau/Aβ43 and t-tau/Aβ42 (AUC 0.81 and 0.72, respectively, *p*-values 0.001 and 0.018, respectively). T-tau/Aβ43 had higher specificity (90%), whereas t-tau/Aβ42 had higher sensitivity (85%). When comparing AUC between sMCI and pMCI subgroups, the t-tau/Aβ43-ratio gave a larger AUC than the t-tau/Aβ42-ratio (*p* = 0.040), but for all other comparisons of Aβ43 and Aβ42, or comparisons of ratios t-tau/Aβ43 and t-tau/Aβ42 between two and two diagnostic groups, there were no significant differences in AUC.

**Table 3:** Diagnostic accuracy of CSF biomarkers at baseline.

	Controls vs. AD	Controls vs. pMCI	Controls vs. sMCI	sMCI vs. AD	pMCI vs. AD	sMCI vs. pMCI
Aβ43	AUC: 0.97 Sens: 93 Spec: 96	AUC: 0.93 Sens: 90 Spec: 100	AUC: 0.75 Sens: 53 Spec: 100	AUC: 0.73 Sens: 86 Spec: 68	AUC: n.s.	AUC: 0.71 Sens: 80 Spec: 68
Aβ42	AUC: 0.96 Sens: 86 Spec: 96	AUC: 0.94 Sens: 85 Spec: 96	AUC: 0.81 Sens: 63 Spec: 92	AUC: n.s.	AUC: n.s.	AUC: n.s.
t-tau/Aβ43	AUC: 0.94 Sens: 93 Spec: 91	AUC: 0.91 Sens: 90 Spec: 96	AUC: 0.72 Sens: 58 Spec: 91	AUC: 0.83 Sens: 79 Spec: 90	AUC: n.s.	AUC: 0.81^∗^ Sens: 75 Spec: 90
t-tau/Aβ42	AUC: 0.95 Sens: 86 Spec: 96	AUC: 0.91 Sens: 90 Spec: 96	AUC: 0.78 Sens: 74 Spec: 87	AUC: 0.78 Sens: 64 Spec: 90	AUC: n.s.	AUC: 0.72^∗^ Sens: 85 Spec: 63

*^∗^For comparison of sMCI vs. pMCI subgroups, the AUC of the t-tau/Aβ43-ratio is larger than the AUC of the t-tau/Aβ42-ratio (*p* = 0.040, DeLong method). AD, Alzheimer’s disease; pMCI, patients with mild cognitive impairment that progressed to AD over 2 years; sMCI, patients with mild cognitive impairment that did not progress to AD over 2 years; Aβ43, amyloid beta 1–43; Aβ42, amyloid beta 1–42; t-tau, total tau; AUC, area under the ROC curve; Sens, sensitivity (%); Spec, specificity (%); n.s., not significant.*

### Longitudinal Biomarker Levels

Longitudinal biomarker data are given in **Table 2**. Means and error bars for CSF Aβ43, Aβ42, t-tau/Aβ43, and t-tau/Aβ42 in all groups at baseline, after 1 year and after 2 years are shown in Supplementary Figures S1A–S1D.

After 1 year, group levels varied for CSF Aβ43 and the t-tau/Aβ43 and t-tau/Aβ42 ratios (all *p* ≤ 0.003), but not for CSF Aβ42. For CSF Aβ43, the t-tau/Aβ43 and t-tau/Aβ42 ratios, there were differences between AD and the sMCI subgroup (all *p* ≤ 0.003), and the sMCI and pMCI subgroups (all *p* ≤ 0.004).

After 2 years, there were variations in group levels for CSF Aβ43 and the t-tau/Aβ43 and t-tau/Aβ42 ratios (all *p* ≤ 0.031), but not for CSF Aβ42. For CSF Aβ43, the t-tau/Aβ43, and t-tau/Aβ42 ratios, there were differences between AD and the sMCI subgroup (all *p* ≤ 0.036), and the sMCI and pMCI subgroups (all *p* ≤ 0.016). No significant differences were found between patients with AD and the pMCI subgroup for any analytes or ratios at either one or 2 years.

**FIGURE 1:**
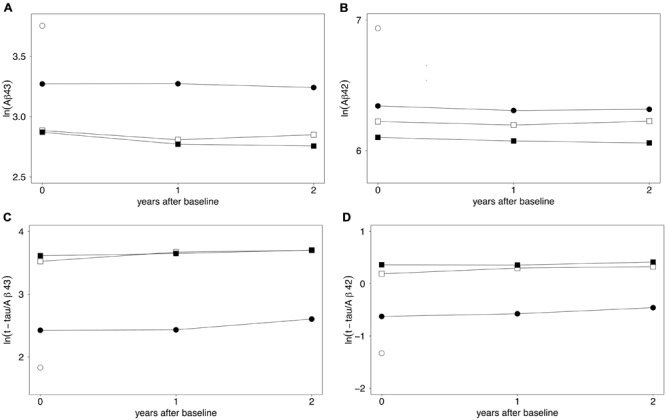
**(A–D)** Longitudinal biomarker levels expressed by a mixed linear model. Estimates of mean log-transformed biomarker levels of **(A)** CSF Aβ43, **(B)** CSF Aβ42, **(C)** t-tau/Aβ43, and **(D)** t-tau/Aβ42 over 2 years are shown. ○ control group (baseline only), ● sMCI, □ pMCI, ■ AD. For clarity, the spread of results is not shown. Means and standard deviations are given in **Table 2**. All analytes were already significantly changed compared to the control level at baseline. Decreased Aβ43 levels over 2 years were found in the AD group (*p* = 0.003). Increased levels over 2 years were found for t-tau/Aβ43 and t-tau/Aβ42 ratios in the sMCI group (*p* ≤ 0.002) and in the pMCI group (*p* ≤ 0.003). Differences between groups at the three time-points are shown in **Table 2** and Supplementary Figures S1A–S1D. Aβ43, amyloid beta 1–43; Aβ42, amyloid beta 1–42; t-tau, total tau; sMCI, patients with mild cognitive impairment that did not progress to Alzheimer’s disease during the 2 years; pMCI, patients with mild cognitive impairment that progressed to AD during the 2 years of study; AD, Alzheimer’s disease.

**Figures 1A–D** show estimated longitudinal biomarker levels for the mixed linear model, for each participant group. The model has not been corrected for age at inclusion or gender, as these factors were not found to significantly affect the model.

CSF Aβ43 showed a decrease from baseline to 1 year after inclusion in both pMCI (*p* = 0.023) and AD groups (*p* = 0.005), but no significant changes were observed from year one to year two after inclusion in these two groups. In the AD group, CSF Aβ43 levels were decreased from baseline to 2 years after inclusion (*p* = 0.003), and levels were stable over 2 years in the sMCI group. For CSF Aβ42, levels were stable over 2 years in all three patient groups.

For the t-tau/Aβ43-ratio, levels were stable over 2 years in the AD group. In the sMCI and pMCI groups, the ratio increased from baseline to 2 years (both *p* = 0.002). For sMCI, the increase was significant from year one to year two (*p* = 0.004), but not from baseline to 1 year after inclusion. For pMCI, the increase was significant from baseline to year one (*p* = 0.010), but not from year one to year two.

For the t-tau/Aβ42-ratio, levels were stable over 2 years in the AD group. In the sMCI and pMCI subgroups, the ratio increased from baseline to 2 years thereafter (both *p* ≤ 0.003). For sMCI, there was an increase from year one to year two (*p* = 0.015), but not from baseline to 1 year after inclusion. For pMCI, there was an increase from baseline to year one (*p* = 0.018), but not from year one to year two.

## Discussion

Patients with aMCI do not necessarily progress to AD. However, being able to distinguish individuals that are at greatest risk of progressing to dementia before brain atrophy becomes so pronounced that symptoms significantly impair cognitive function is an important goal for biomarker research. In the present study, half the patients initially diagnosed with aMCI progressed to AD during the 2 years of study. This pMCI subgroup was therefore comparatively homogeneous, given that patients were all in a prodromal phase, close to the development of Alzheimer dementia, which probably explains why the pMCI and AD groups could not be separated by any biomarker or combination of biomarkers at baseline. The ability to separate out such patients within the total aMCI group will be urgent once more effective treatment becomes available to prevent, or at least slow, disease progression.

At baseline, as well as after one and 2 years, CSF Aβ43 with or without t-tau in the equation separated patients with aMCI not progressing to AD within the 2 years of study from patients that did progress to AD, as well as from those diagnosed with AD at baseline. No such significant separation was found for CSF Aβ42 alone. Therefore, and because t-tau/Aβ43 also seemed to be at least as good as t-tau/Aβ42 for diagnostic accuracy at baseline in this study, CSF Aβ43 could be a useful biomarker for identifying patients with aMCI at greatest risk of AD. However, even though t-tau/Aβ43 in the present study had a slightly and significantly larger area under the ROC curve for distinguishing between the two aMCI subgroups at baseline, t-tau/Aβ42 was better for identifying patients in an early stage of AD (higher sensitivity).

To the best of our knowledge, this is the first longitudinal study of CSF Aβ43 in connection with aMCI and AD. A significant reduction in the CSF Aβ43 level over the 2 years following baseline was observed in the AD group, whereas no significant longitudinal changes in CSF Aβ43 levels were observed in the sMCI and pMCI groups. The longitudinal changes during the 2 years of study were substantially less in all patient groups compared to the concentration difference between the control group and patient groups at baseline. This is an indication that the reduction of CSF Aβ43 is most pronounced during the preclinical phase of disease, which may take place slowly over many years. It is certainly clear that changes in CSF Aβ43 concentration over the 2 years of our study are slow (according to our data no more than 0.5–1% annually). Since the standard deviation was relatively small in the AD group, the reduction was significant. However, this was not the case in the pMCI or sMCI subgroups where standard deviations were larger, and indeed there was hardly any change over 2 years in the sMCI subgroup.

The sMCI subgroup was probably heterogeneous, and although it contained individuals who have converted to AD since the end of the study (data not shown), it probably also contains individuals with control levels that will never convert to any neurodegenerative disease (Nettiksimmons et al., 2014; Tifratene et al., 2015). Overall, the annual small change in such a group is likely to be difficult to detect statistically.

Despite the heterogeneity of the sMCI subgroup, baseline mean levels of CSF Aβ43 (but not CSF Aβ42) distinguished significantly between the two aMCI subgroups, even though the groups were small. Contrary to CSF Aβ43, CSF Aβ42 levels were stable from baseline to 2 years in all three patient groups, in agreement with an earlier longitudinal study showing that CSF Aβ42 levels were stable over 4 years in MCI patients (Mattsson et al., 2012), and in agreement with results showing fully reduced CSF Aβ42 levels several years before dementia symptoms appear (Buchhave et al., 2012).

CSF Aβ43 and Aβ42 were highly correlated in all four participant groups at baseline. Aβ43 and Aβ42 are suggested to be produced from different routes of enzymic cleavage, where three amino acids are cleaved off the peptides Aβ48 and Aβ49 in two steps to produce Aβ42 and Aβ43, respectively (Qi-Takahara et al., 2005). The close correlation of Aβ42 and Aβ43 in the present study supports a connection between the two synthetic pathways, as shown earlier (Kakuda et al., 2012).

This is a small study with groups of limited size. In an earlier study of similar size that compared AD patients with controls, CSF Aβ43 was found to have similar specificity (97%) but much lower sensitivity (52%) and lower AUC (0.77) than in the current study (Bruggink et al., 2013). Such differences between studies arise easily when comparing small groups, and the present data should therefore be confirmed in a larger material. However, one of the main strengths of the present study is that all patients were diagnosed by a single neurologist, ensuring consistency of diagnoses between and within groups. Additionally, diagnoses were confirmed by a second neurologist.

## Conclusion

Our findings suggest that CSF Aβ43, either alone or in a ratio with CSF t-tau, may be a useful additional discriminator for patients with aMCI that will progress to AD within a short period of time.

## Author Contributions

CL planned and performed the laboratory work, analyzed data, and wrote the manuscript. SS performed the clinical examination of all study participants together with GG. AS, IM, and GBe planned and performed the laboratory work. IB and ØS advised on statistical analysis. SS, GBr, and LW contributed to the conceptual design of the study, and supervised the project. All authors contributed to critical revision and finalization of the manuscript.

## Conflict of Interest Statement

The authors declare that the research was conducted in the absence of any commercial or financial relationships that could be construed as a potential conflict of interest.
