# Supplementary material for: Cerebrospinal Fluid Levels of Amyloid Beta 1–43 in Patients with Amnestic Mild Cognitive Impairment or Early Alzheimer’s Disease: A 2-Year Follow-Up Study
Source: Front Aging Neurosci. 2016 Mar 1;8:30. doi: 10.3389/fnagi.2016.00030 (PMC4772322; doi:10.3389/fnagi.2016.00030)
Supplement: Supplementary file 1 [file Presentation_1.PDF]

Supplementary Material

Cerebrospinal fluid levels of amyloid beta 1-43 in patients with amnestic mild cognitive impairment or early Alzheimer's disease: a 2-year follow-up study

Camilla Lauridsen[1], Sigrid Botne Sando[1,2], Adiba Shabnam[1], Ina Møller[2], Guro Berge[1], Gøril Rolfseng Grøntvedt[1,2], Inger Johanne Bakken[3], Øyvind Salvesen[4], Geir Bråthen[1,2], Linda Rosemary White[1,2,*]

*Corresponding author: Linda R. White, linda.white@ntnu.no

Supplementary Figure S1A-D. Biomarker concentrations in cerebrospinal fluid at baseline, after one year and after two years. Means and error bars representing +/- 1 standard deviation are given for all four participant groups. **(S1A)**: Aβ43, **(S1B)**: Aβ42, **(S1C)**: t-tau/Aβ43, **(S1D)**: t-tau/Aβ42. Values for mean ± standard deviation are given in Table 2. Statistical analyses of group differences were performed on log-transformed values (ANOVA followed by LSD post hoc test). [#]Control group levels at baseline were significantly different from all three patient groups (all p≤0.007). *p<0.05, **p<0.01, ***p≤0.001. Aβ43: amyloid beta 1-43, t-tau: total tau, sMCI: patients with mild cognitive impairment that did not progress to Alzheimer's disease over two years, pMCI: patients with mild cognitive impairment that progressed to AD over two years, AD: Alzheimer's disease, SD: standard deviation.

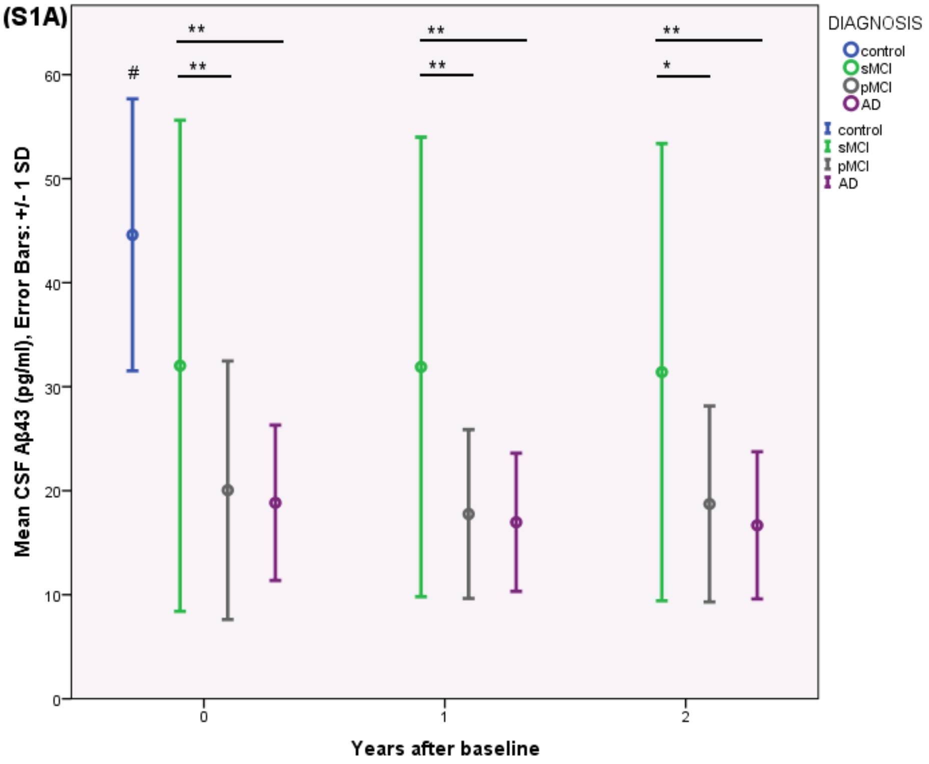

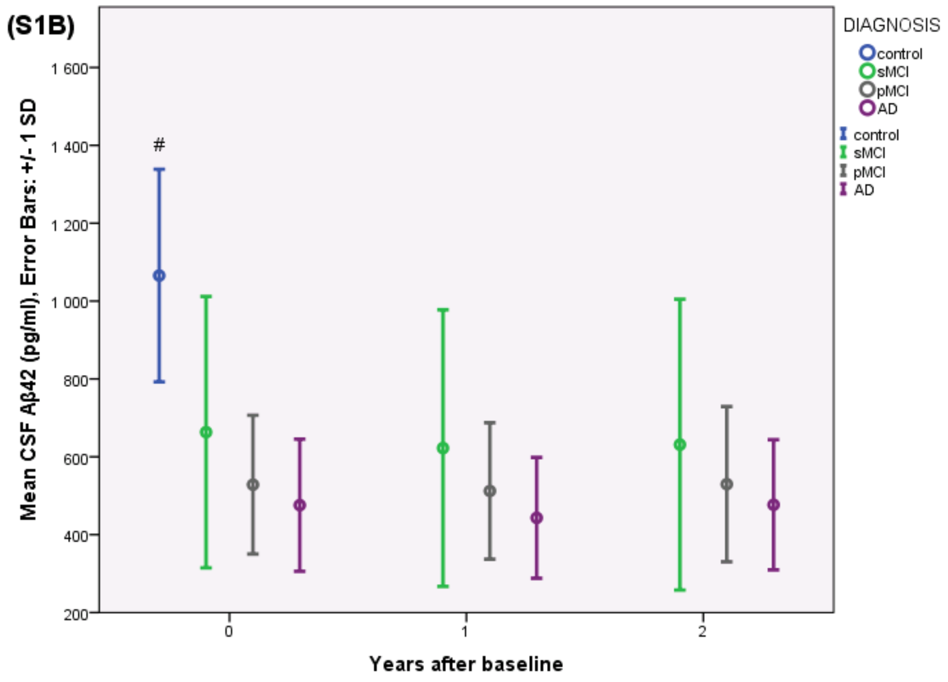
(S1B)
DIAGNOSIS
control
sMCI
pMCI
AD
Mean CSF Aβ42 (pg/ml), Error Bars: +/- 1 SD
1 600
1 400
1 200
1 000
800
600
400
200
#
0
1
2
Years after baseline

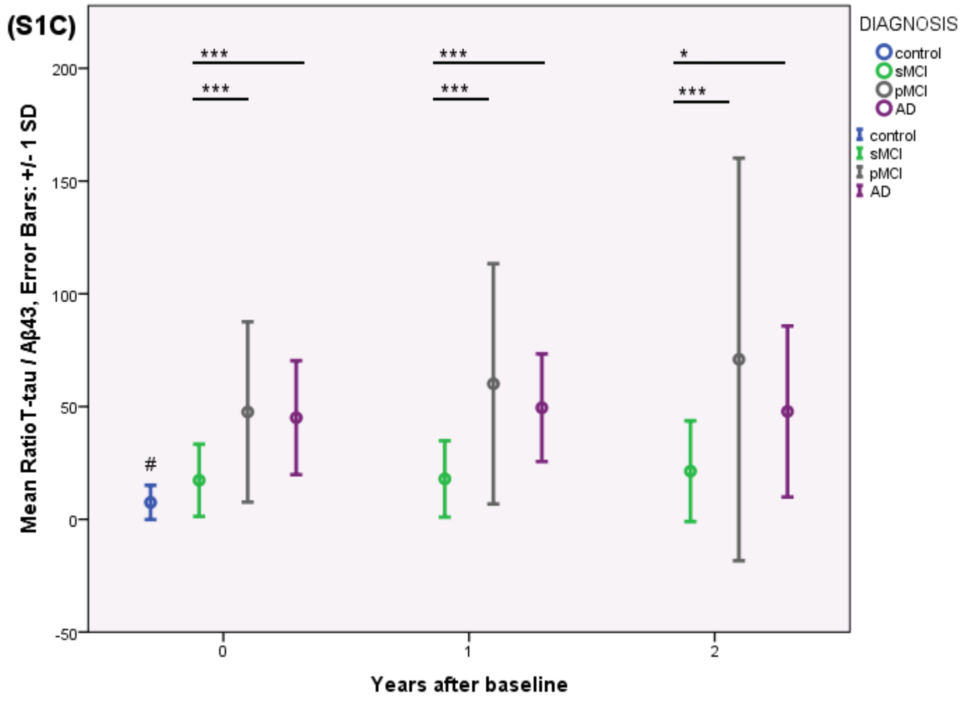
(S1C)
DIAGNOSIS
control
sMCI
pMCI
AD
Mean Ratio T-tau / Aβ43, Error Bars: +/- 1 SD
200
150
100
50
0
-50
***
*
#
0
1
2
Years after baseline

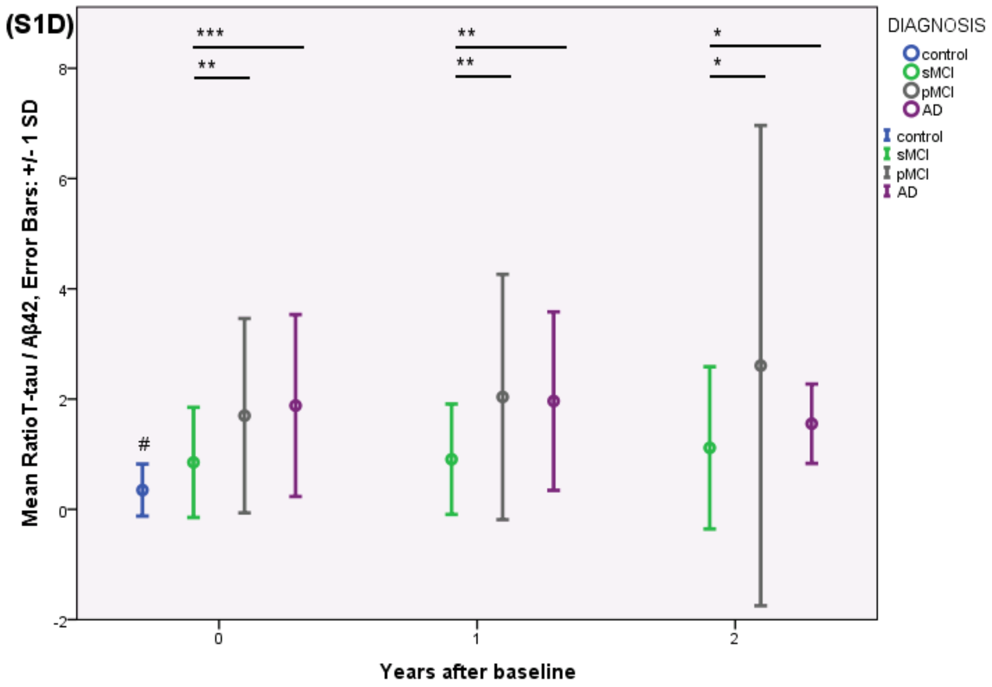
(S1D)
***
**
**
**
*
*
#
DIAGNOSIS
control
sMCI
pMCI
AD
control
sMCI
pMCI
AD
Mean Ratio T-tau / Aβ42, Error Bars: +/- 1 SD
8
6
4
2
0
-2
0
1
2
Years after baseline
